# CoPub Mapper: mining MEDLINE based on search term co-publication

**DOI:** 10.1186/1471-2105-6-51

**Published:** 2005-03-11

**Authors:** Blaise TF Alako, Antoine Veldhoven, Sjozef van Baal, Rob Jelier, Stefan Verhoeven, Ton Rullmann, Jan Polman, Guido Jenster

**Affiliations:** 1 Department of Molecular Design & Informatics, Organon NV, P.O. Box 20, 5340 BH Oss, The Netherlands; 2 Department of Urology, Erasmus MC, P.O. Box 1738, 3000 DR Rotterdam, The Netherlands; 3 Department of Genetics, Erasmus MC, Rotterdam, The Netherlands; 4 Department of Medical Informatics, Erasmus MC, Rotterdam, The Netherlands

## Abstract

**Background:**

High throughput microarray analyses result in many differentially expressed genes that are potentially responsible for the biological process of interest. In order to identify biological similarities between genes, publications from MEDLINE were identified in which pairs of gene names and combinations of gene name with specific keywords were co-mentioned.

**Results:**

MEDLINE search strings for 15,621 known genes and 3,731 keywords were generated and validated. PubMed IDs were retrieved from MEDLINE and relative probability of co-occurrences of all gene-gene and gene-keyword pairs determined. To assess gene clustering according to literature co-publication, 150 genes consisting of 8 sets with known connections (same pathway, same protein complex, or same cellular localization, etc.) were run through the program. Receiver operator characteristics (ROC) analyses showed that most gene sets were clustered much better than expected by random chance. To test grouping of genes from real microarray data, 221 differentially expressed genes from a microarray experiment were analyzed with CoPub Mapper, which resulted in several relevant clusters of genes with biological process and disease keywords. In addition, all genes versus keywords were hierarchical clustered to reveal a complete grouping of published genes based on co-occurrence.

**Conclusion:**

The CoPub Mapper program allows for quick and versatile querying of co-published genes and keywords and can be successfully used to cluster predefined groups of genes and microarray data.

## Background

High throughput microarray analysis has made it possible to analyze the mRNA expression of most if not all human genes simultaneously [1,2]. The data generated from these analyses are overwhelming since hundreds of interesting differentially expressed genes can be identified in a single assay. Knowledge on expression levels of genes in different systems is useful, but does not directly answer biologically relevant questions, such as: What is the gene function? Where is the gene located within the genome? Where is the protein located within the cell? Most important is the answer to the question whether genes identified in microarray experiments have something in common, such as, are multiple genes part of a single biological pathway or proteins part of a protein complex? The public database which contains much of the relevant information to answer these questions is MEDLINE. Therefore, mining the MEDLINE database for all information on a set of genes of interest to extract and evaluate their co-occurrences with biological keywords and other genes, could reveal biologically relevant pathways [3-6].

The most widely used methodology to identify genes and proteins in text is by thesaurus-based concept extraction. Using a predefined gene name list, text phrases are compared to the thesaurus for matching. Complications for gene name thesauri are variations in full name spelling, use of abbreviations (gene symbols), the large number of synonyms (different name but same gene) and homonyms (same name but meaning different genes or unrelated concepts) [7,8]. Particularly homonyms in the form of abbreviations and acronyms create a serious problem of false positive assignment of a gene to a particular concept [9-13]. A complementary approach for gene/protein identification is "named entity recognition" in which a program learns to recognize concepts from text [14-16]. Due to the enormous synonym and homonym problems, named entity recognition encounters difficulties in achieving high performance gene name identification. A next step in text mining is linking of different concepts (such as gene names and keywords) that are identified. In the simplest method, co-occurrence of two concepts within the document can be used as an indication of linkage. Extensions of co-occurrence can include (i) the number of times a concept is found, (ii) how close concepts are to one another, such as, within a single sentence, and (iii) not just two, but the weighed combination of all concepts within a document. More sophisticated fact extraction methods can also retrieve information on the type of relationship between two concepts. Natural language processing (NLP) grammatically parses whole sentences to identify verbs and other connecting phrases that describe the correlation between concepts [3,4,6,17]. A third step in text mining takes linked concepts and groups them according to their co-occurrence and relationships. Again, this can be performed by simple clustering of the co-occurrence of pairs of concepts as well as complex multi-dimensional classification using weighed concept combinations [18,19]. This type of clustering of, for example, differentially expressed genes from a microarray experiment, can disclose, summarize, and visualize published knowledge, but can also be utilized for novel information discovery [5,20]. Although progress is being made in higher order literature processing, text mining applications in the field of genomics are mainly thesaurus and co-occurrence based. Such programs and methods to identify potential functional correlations between genes have been described [21-33]. Each of these applications has its unique advantages and limitations, showing the broad range of needs for text mining as well as the numerous extraction, linking, and discovery methods feasible.

We set out to create a well annotated and curated open source gene list including full names, symbols and aliases and a regular expression-based search method to identify genes in text databases such as MEDLINE. In addition to the gene thesaurus, specific keyword lists were generated for co-occurrence analyses. For each concept, PubMed identifiers (IDs) from MEDLINE documents containing the concept were extracted, all gene-gene and gene-keyword co-occurrence pairs identified and stored in a database for fast co-occurrence retrieval. This database can be mined using single or batches of concepts to retrieve co-occurrences that form the input in clustering programs to group genes and keywords according to their similarity in co-publications. The program, database and all thesauri are freely available and can be adapted to include updates, new thesauri, and search methods.

## Implementation

### Human gene thesaurus

**Figure 1:**
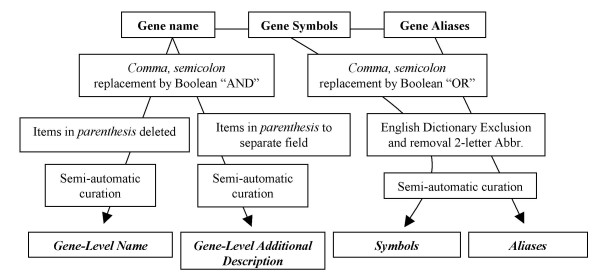
Flow diagram of the processing and curation of the gene names, symbols and aliases. Gene names, symbols and aliases were retrieved from Affymetrix HG_U95 / HG_U133 and the HUGO databases.

A human gene thesaurus was compiled from the Affymetrix HG_U95 / HG_U133 and HUGO gene annotations (HG_U95 / HG_U133 annotation files from 2002) [34,8] (Table 1). In total, 15,621 annotated genes were included of which most gene descriptions consist of one or more full names, the gene symbol, and their aliases. The typical HUGO and Affymetrix full gene name descriptions contain commas, semicolons and often alternative names in parenthesis, which makes this description an inadequate direct search term. Full names were processed by replacing the commas and semicolons with the Boolean "AND" operator (Figure 1). All terms included in parentheses were deleted from "gene-level name" and placed in a separate field named "gene-level additional description". Both fields were semi-automatically curated to remove common words (such as protein, family, hypothetical, functional, human, tissue, yeast, etc), misspellings, and insert Boolean "OR" in case synonyms are described. From gene symbols and aliases fields, commas and semicolons separators were replaced by the Boolean "OR" operator. Two-letter symbols and aliases were removed from the thesaurus and all other abbreviations were compared to an English dictionary [35] to remove common English words (such as "AND", "CELL", etc.). The Microsoft Excel spreadsheet program was used for generating and curating gene thesaurus files and, as described by Zeeberg et al [36], conversion problems were encountered and when identified, manually corrected.

Semi-automatic stemming was performed on "gene-level name" and "gene-level additional description" fields by removing numbers, letters, and phrases like "alpha", "member", "type", "class", etc. This resulted in a stem-level gene name description. Although the current version of CoPub Mapper does not take this stem-level into account, these fields are part of the gene thesaurus and freely available.

### Keyword thesauri

In total, five different keyword thesauri were compiled including the Gene Ontology "biological process", "cellular component", and "molecular function", as well as "diseases" and "tissues" (Table 1). In the disease thesaurus, commas were replaced with the Boolean "OR" operator. All keyword databases were manually curated to remove terms too specific or too common.

### MEDLINE concept extraction and curation

The full MEDLINE baseline XML files (until January 2004) were obtained from the National Library of Medicine [37], extracted to small text files containing title, abstract and substances using BioPerl API. The title, substance and abstract fields from MEDLINE records from 1966 to January 2004 were searched for the presence of different case-insensitive gene and keyword concepts using Perl compatible regular expressions (PCRE). For the gene-level name descriptions the characters "] [.-)(,:;" and space were allowed preceding and following the gene-level name description and also an optional "s" was permitted to follow the name. Any space in the gene-level name description was allowed to be a space or a dash. The same regular expressions were applied to the gene name stem-level descriptions, except that, the description could also be followed by any single letter or a number between 0 and 99. Gene symbols and aliases could be preceded and followed by the characters "] [.-)(,:;" and space. After the first two characters, the presence of a dash was allowed in between the characters of the symbols and aliases (to take, for example, both "bcl2" and "bcl-2" into account). The concepts of the keyword files could be preceded and followed by the characters "][.-)(,:;" and space. In addition, "s" and "'s" were allowed to follow the disease concept. As for the gene-level name descriptions, a dash was allowed to be present between the words of a keyword concept. Per annotated gene or keyword, the PubMed IDs of MEDLINE records in which the concept was identified were stored in a MySQL database.

In order to identify potential problem concepts, 50 genes and 50 keywords with the highest number of PubMed IDs were manually inspected and curated if appropriate. In addition, a random selection of genes and all keywords that gave less than 2 MEDLINE hits were examined and this evaluation was used to optimise the thesauri and regular expressions search strategy described above.

To address the homonym issue, a correction was made for possible discrepancies between a parenthesised gene symbol and its expected name. All abbreviations in parenthesis in MEDLINE abstracts were retrieved in combination with 4 preceding words. In total, 1,105,669 MEDLINE records were identified where the abbreviation matched a gene symbol or alias. For all these records, 4 words preceding the abbreviation were compared to the gene-level name description of that particular gene. If none of the words resembled partly the gene name, the PubMed ID was removed from that particular gene's PubMed ID list. Using this method, 603,580 records were deleted from the gene hit database resolving part of the gene-unrelated concept homonym problems. Manual inspection of 173 random records revealed that, extrapolated, 79 % of the 603,580 records was correctly removed, while 7 % of the 502,089 non-removed records should have been deleted.

In our examination of genes with the highest number of PubMed IDs and our first CoPub Mapper analyses, we noticed a distinct contamination of records identifying gene symbols and aliases by abbreviation used for cell lines (such as PC3 which is an alias for 3 genes as well as a prostate cancer cell line). Since full names of cell line abbreviations are rarely put in writing, the homonym correction did not eliminate these discrepancies. A list of cell line names was retrieved [38] and gene symbols and aliases that fitted a cell line name were further processed. From 106 genes that included one of the cell line homonym names, all MEDLINE records were deleted in which the cell line name was mentioned without the presence of the stem-level gene name. In total, 100,213 PubMed IDs were eliminated. A manual inspection of 78 randomly chosen records showed that 87 % were correctly removed.

### Database set-up and CoPub Mapper program

A file was generated that contains a unique query ID and the probeset IDs, UniGene (combination of Aug 2002 and Oct 2003 builds) and RefSeq identifiers for each of the individual 15,621 entries in the gene thesaurus (alias_affygene). In addition, a file with the gene name, symbol and aliases and unique query ID was created (query_affygene).

The retrieved PubMed IDs from each field (gene names, symbols and aliases) of the 15,621 unique gene thesaurus query IDs were non-redundantly combined into a MySQL database (lit_affygene) and a separate data-file (litstat_affygene) in which the number of PubMed IDs per query was counted. Furthermore, the PubMed IDs from the keyword thesauri were per concept stored (query_*keyword*, lit-*keyword *and litstat_*keyword*). Per gene-gene pair and gene-keyword pair, overlaps in PubMed IDs were identified and separately stored in the database (pair_*keyword*_affygene). From these paired files, a pairstat file was generated containing the number of PubMed IDs of each concept, the number of overlapping PubMed IDs between the two concepts and a relative score. The relative score is based on the mutual information measure and was calculated as

S = P_AB_/P_A _* P_B_

in which P_A _is the number of hits for concept A divided by the total number of PubMed IDs, P_B _is the number of hits for concept B divided by the total number of PubMed IDs, and P_AB _is the number of co-occurrences between concepts A and B divided by the total number of PubMed IDs. The relative score is produced as a log10 conversion and in the batch search option in a 1–100 scaled log10 conversion:

R = ^10^log S

and the scaled log transformed relative score:

R' = 1 + 99 * (R - Rmin) / (Rmax - Rmin)

where Rmin and Rmax are the lowest and highest R values in each pairstat file, respectively.

The CoPub program was generated in Python and runs as a web-based application (CGI script). The text output of a batch search can be saved and imported into a clustering program such as Cluster [39] and SpotFire (Spotfire, Göteborg, Sweden). The HTML output of "number of hits", "relative score", and batch search results are hyperlinked to the MEDLINE database at the European Bioinformatics Institute [40] for direct manuscript retrieval.

### Performance evaluation using ROC (receiver operating characteristics) curves

In order to investigate whether the CoPub Mapper output could group genes according to their MEDLINE co-occurrence profile, 8 different groups of genes were defined based on common gene ontology (GO) terms [41], the BRCA1 BioCarta pathway [42], or a microarray experiment (Table 2). In the UniGEM V microarray experiment, the gene expression profile of prostate stroma cells was compared to prostate epithelial cells [43]. A set of 28 annotated genes, higher expressed in epithelial cells as compared to stromal cells (more than 2-fold) were randomly selected.

**Figure 2:**
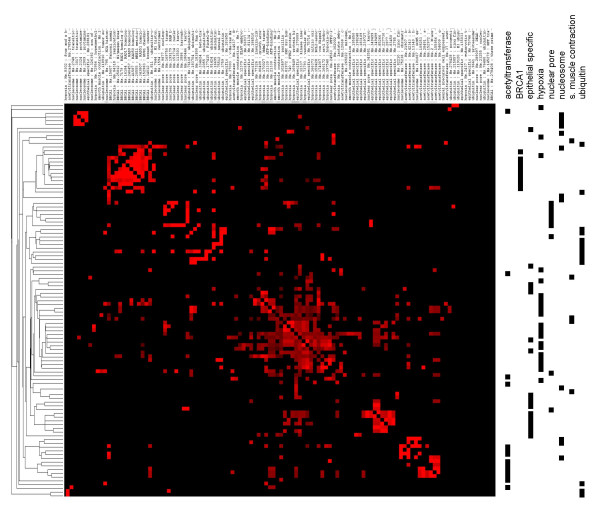
Clustered view of gene co-occurrences among a collection of 8 groups of selected genes. Of the 150 genes, the relative scores of co-occurrences were calculated and clustered using hierarchical clustering. A co-occurrence was only taken into account when at least two articles mention the gene-gene pair. Using this criterion, 45 genes did not co-publish with any of the other 149 genes. To which group (Table 2) a gene belongs to is indicated in the right part of the figure. Image contrast in TreeView was set at 50. Scaled (1–100) relative scores are represented in a red spectrum with bright red being the highest score. A relative score of zero or no score are in black.

The 150 genes from the eight selected gene groups are pooled into one set. The selected genes were entered into CoPub Mapper to generate the co-occurrence matrix of relative scores of genes versus genes and genes versus the 5 different keyword thesauri. Relative scores were only generated in case more than 2 co-publications occurred per concept-concept pair. The genes versus genes matrix was hierarchical clustered and visualised using Cluster and TreeView [39] (Figure 2).

For a systematic evaluation of performance we applied Receiver Operating Characteristics (ROC) graphs and the area under the ROC curve (AUC) as an outcome measure. To use this method all genes from the 8 subgroups are pooled into one set. To calculate an AUC for every gene we used the following procedure. A gene from the pooled set is selected as a seed. The seed is paired with all other genes in the set and non-centered Pearson correlation coefficients are calculated based on their co-occurrence profiles. The co-occurrence profile is one row of the co-occurrence matrix under investigation. The genes are ordered by their correlation coefficients, with the highest value at the first rank. To generate a ROC curve, the obtained ranking of the genes is viewed as the outcome of a classifier. For a seed, genes from the same subgroup are called positives and all other genes are called negatives. ROC curves are two-dimensional graphs in which the true-positive (TP) rate is plotted against the false-positive (FP) rate. The TP rate is defined as correctly classified positives divided by all positives. The FP rate is defined as incorrectly classified negatives divided by all negatives. While running down the list, for every rank the true and false positive rate are calculated, by taking all encountered genes to be classified as positive and all not yet encountered genes as negative. The AUC of the ROC curve is calculated. The procedure is repeated until an AUC has been calculated for every gene in the pooled set. An average AUC is calculated per subgroup. The AUC measure varies between 0 and 1. Random ordering gives an AUC of 0.5 and an AUC of 1 represents perfect ordering, i.e. all positives are at the top of the list with no negatives in between, indicating perfect co-occurrence clustering of the genes in the subgroup [44].

## Results

### Validation of CoPub Mapper co-occurrence profiling

**Figure 3:**
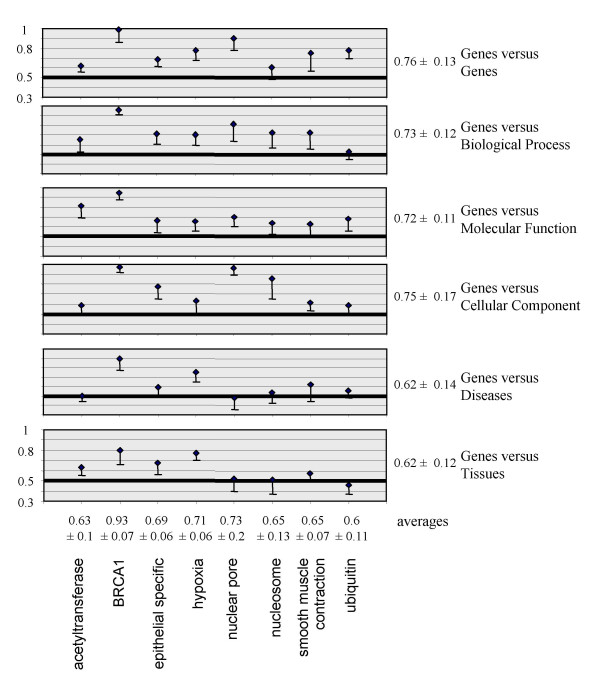
Receiver operating characteristics (ROC) of the 8 selected groups of genes to quantify their coherence upon clustering of literature co-occurrences. Co-occurrences of the 150 genes were determined with the genes themselves, or the 5 different keyword thesauri. A co-occurrence was only taken into account when at least two articles mention the gene-gene or gene-keyword pair. The co-occurrence matrixes were Pearson correlation clustered and the distances between genes determined. For each gene, it was determined whether the next closest clustered gene was a group member. Genes from the same group were scored as true positive and any other gene as false positive to generate a ROC curve. For each gene, the area under the ROC curve (AUC) was determined and the median of all the group members per group ± SD depicted. Scaling is from an AUC of 0.3 to 1. An AUC of 0.5, representing a random ordering is highlighted with a thick line.

To validate the usefulness of the CoPub Mapper output, we evaluated how well genes with known relations could be grouped according to their MEDLINE co-occurrence profile. As shown in Figure 2, partial clustering of the initial 8 groups occurred upon their gene-gene co-occurrence profile evaluation. To quantify this grouping, ROC (receiver operating characteristics) curves were generated and the AUCs (Area Under Curve) for each gene calculated. In Figure 3, the median AUCs ± SD of the genes per group are depicted. Most of the 8 groups and in particular the BRCA1-associated genes clustered well together in the gene-keyword comparisons (median AUC of 0.93 ± 0.07). The ubiquitin-associated genes performed worst (median AUC of 0.6 ± 0.11). With respect to the thesaurus selection, the overall clustering of the 8 groups using the "genes versus genes self" comparison, performed best with an average AUC of 0.76 ± 0.13. The "genes versus diseases" and "genes versus tissues" comparisons were for many of the 8 groups not resulting in clustering higher than expected by random chance. In other words, from co-publication analysis of genes with disease or tissue keywords, the commonality between the genes, as defined by the 8 groups, could rarely be traced (Figure 3). As shown in Table 2, six groups of genes were selected based on gene ontology keywords, using two from each of the annotation trees (biological process, molecular function, and cellular component). As expected and without exception, the AUC of the 6 groups of genes was higher using their corresponding GO-derived thesaurus compared to using the other two GO-derived thesauri. For example, the molecular function annotated group of "acetyltransferases" was clustered best using the "genes versus molecular function" co-publication comparison (AUC of 0.81 as compared to 0.65 using the biological process thesaurus and 0.59 using the cellular component thesaurus). This shows that the selection of keywords for co-occurrence analysis is an important determinant in optimal text-based grouping of genes.

### Microarray analysis using CoPub Mapper

In order to validate the CoPub Mapper program with real microarray data, a set of differentially expressed genes was selected from a comparison between ovaries of healthy women and women suffering from Poly Cystic Ovary Syndrome (PCOS) [45]. PCOS is characterized by a combination of chronic anovulation, hyperandrogenism and cysts in ovaries and is the most common cause of anovulatory infertility. Also hyperinsulinemia and obesity can be observed in many PCOS patients [46,47].

**Figure 4:**
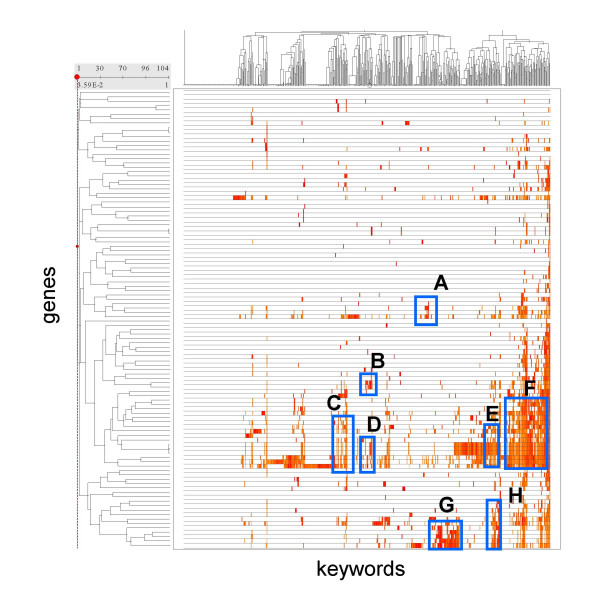
Hierarchical clustering of literature co-occurences of 104 genes (rows) versus 761 biological processes and diseases (columns). A co-occurrence was only taken into account when at least three articles mention the gene-keyword pair. Hierarchical clustering of CoPub Mapper results using genes differentially expressed in PCOS ovaries. From 221 regulated genes 104 genes contain a gene name, symbol or alias and produce a gene-keyword pair with biological processes or diseases. 104 modulated genes returned 761 keywords denoting biological processes or diseases. Hierarchical clustering was performed using Spotfire using the Complete Linkage method and Correlation as Similarity Measure. Several subclusters were identified shown here with blue boxes; between parenthesis the number of genes in a cluster. A: PCOS, Obesity, Insulin Resistance (4); B & D: Gametogenesis (5&8); C: Cell adhesion, Angiogenesis (19); E & H: Immune response, Inflammation (14&11); F: Cancer, Cell growth, Differentiation (32); G: Inflammatory diseases (6).

A set of 230 dysregulated DNA fragments representing 189 genes were used as input for CoPub Mapper (see Table 1 in [45]). Gene-keyword pairs were obtained from biological processes and diseases. Relative scores were only generated in case 3 or more co-publications occurred per gene-keyword pair. From these 189 genes, 104 were annotated and had at least 3 co-publications with one of the keywords. Resulting matrices were exported as text files and opened and merged in Spotfire. Hierarchical clustering was used to group genes and keywords. Figure 4 shows that subsets of genes form clusters with subsets of biological processes and diseases. Zooming in on these clusters confirms the relation of certain genes with e.g. PCOS, diabetes, obesity, gametogenesis, immune response. Characterization of all clusters revealed known and unknown relations of these PCOS dysregulated genes with biological processes and diseases.

### Single Gene-Keyword extraction

The CoPub Mapper includes an option to query the database for all genes and keywords co-published with a single gene of interest. In addition, a keyword of interest can be selected and all genes with 2 or more co-occurrences can be extracted. As examples, the top ten genes (Table 3) and top ten diseases (Table 4) co-published with the androgen receptor are shown. An assessment of the 2 lists identified the puromycin-sensitive aminopeptidase gene (NPEPPS) as an example of a homonym (Table 3, fourth gene). The PSA alias of NPEPPS is mainly used to specify prostate specific antigen. The prostate specific antigen gene (KLK3) is regulated by the androgen receptor and correctly found many times to be co-published with the androgen receptor (Table 3, second gene). Due to the homonym curation described in the Systems and Methods section, the number of co-occurrences of the androgen receptor with NPEPPS (246) is lower than with KLK3 (414). Before homonym curation, NPEPPS and KLK3 had 634 and 635 co-publications with the androgen receptor, respectively. The top ten list of diseases co-published with the androgen receptor (Table 4) is a near perfect reflection of the known diseases associated with androgen receptor activity and aberrations.

In Table 5, the top ten genes are listed that are most often co-published with the keyword "prostate cancer". Again, the incorrect identification of NPEPPS in 4507 MEDLINE entries is due to the PSA homonym.

### Meta-analysis: all genes versus keywords

**Figure 5:**
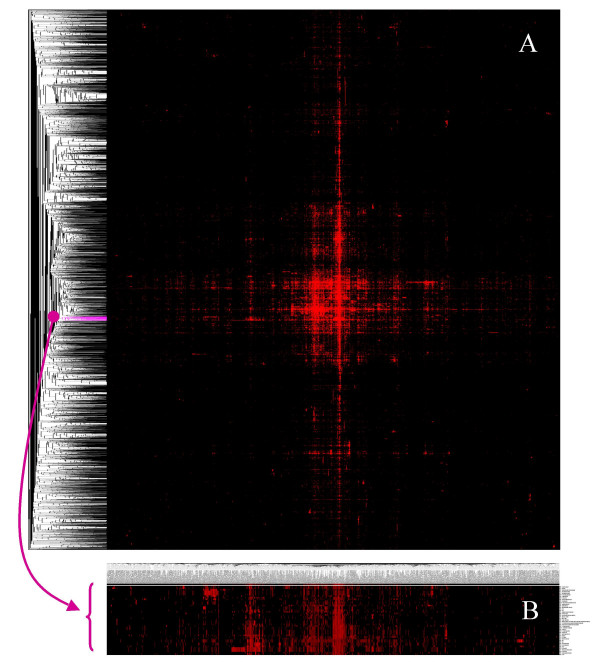
Hierarchical clustering of literature co-occurrences of 5626 genes (rows) versus 1275 diseases (columns). A co-occurrence was only taken into account when at least two articles mention the gene-disease pair. Each gene had to have at least once a high (1–100 scaled) relevance score of >46. **A**: Overview of all 5626 genes and 1275 diseases. **B**: Enlargement of a small subsection of genes showing the amount of detail present in the CoPub Mapper analysis.

In order to provide a summary of all gene-keyword co-occurrences, CoPub Mapping was performed using all 15,621 annotated genes as input in the different gene-keyword thesauri co-occurrence comparisons. Relative scores were only computed if in at least two articles a co-occurrence was observed. Elimination of single gene-keyword co-publications was carried out to eradicate non-reproduced findings and to make the large matrices manageable. A second selection was made to eliminate genes which included only low relative scores. Many genes have multiple co-publications with very common keywords such as "cancer" (disease thesaurus), "cytoplasm" (cellular component thesaurus), etc. If not functionally relevant, these co-occurrences have typically a low relevance score. Genes with only low relevance scores were eliminated by removing those genes that did not have 1 or more scaled relevance scores of more than a threshold (between 39 and 52) in which 20 % of genes were eliminated. The hierarchical clustered genes-diseases co-publication matrix is displayed in Figure 5. 5626 genes (rows) versus 1275 diseases (columns) were grouped according to their co-publication profiles. The enlarged section shows the amount of detail present in the matrix (Figure 5B). The vertical lines in the matrix are caused by co-publication of almost all genes with very common disease keywords such as "cancer", "neoplasm", and "carcinoma". Horizontal lines are genes co-published with many diseases, such as "insulin", "interleukin 6", and "keratin 3A". If low relevance scores are masked by hiding values below 30 in TreeView or SpotFire, these streaks become less prominent.

**Figure 6:**
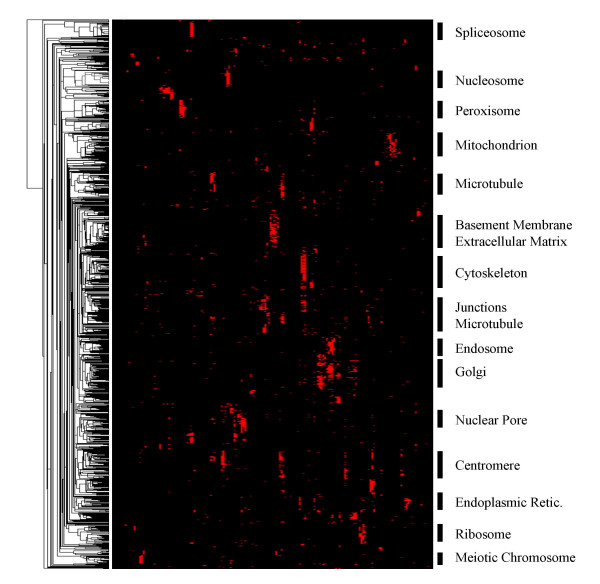
Hierarchical clustering of literature co-occurrences of 1135 genes (rows) versus 177 cellular components (columns). A co-occurrence was only taken into account when at least three articles mention the gene-cellular component pair. Each gene had to have at least twice a high (1–100 scaled) relevance score of >50. Relative scores of less then 50 were masked in the TreeView program. Some of the cellular component concepts responsible for clustering of genes are indicated.

Clustering and visualisation of only highly significant co-occurrences will result in discrete groups of genes and keywords as shown in Figure 6. Stringent selection criteria were implemented including: (i) each gene had to be co-published with at least two different keywords with a relevance score of more than 50, and (ii) a co-occurrence must have been described in at least 3 publications per gene-keyword combination. From the 10,203 genes co-occurring with cellular component keywords, 1135 genes were retrieved using the stringent selection criteria mentioned above. As expected, these genes were clustered according to well-known cellular components of which some examples are depicted (Figure 6).

## Discussion

With the implementation of high-throughput technologies in many fields of research, problems have shifted from data gathering to data comprehension. Linking data from different sources, such as microarray expression data to biomedical text corpora, can assist in the disclosure, summary, and visualisation of knowledge. This is particularly valuable when from high throughput data, only a few items can be selected for further detailed low-throughput examination. Co-occurrence analysis of concepts using the MEDLINE literature database, is an effective tool to extract and categorize published knowledge. CoPub Mapper output was successfully used to cluster predefined groups of genes and resulted in a commonsensical clustering of PCOS microarray data. In addition, CoPub Mapper uncovered relationships between genes using single concept searches and provided an overall gene-keyword clustered summary of the literature. One obvious limitation of gene-driven text mining is the incomplete study and publication of all human genes. Out of approximately 30,000 human genes, we included 15,621 annotated genes of which 10,700 were mentioned at least once and 9,769 at least twice in MEDLINE. The use of human gene names, symbols and aliases does not necessarily mean a human-specific literature search. Many gene names and symbols are shared by other species as well.

The main advantages of CoPub Mapper above most other co-publication programs, are its modularity of keyword databases and the pre-calculated co-occurrences. Based on the results from the predefined groups of genes, the choice of keyword database made a substantial difference in clustering efficiency as determined by AUC calculations. Utilisation of a single joint thesaurus could counteract clustering due to inclusion of irrelevant non-discriminating keywords. Another illustration that keyword selection is an important issue, is the prevalence of common keywords such as "cancer" (disease), "membrane" (cellular component), "metabolism" (biological process), "receptor" (molecular function), and "blood" (tissue). These keywords are co-published with nearly any gene of interest and were identified using CoPub Mapper. Although the relative score is generally low, these co-occurrences will influence the clustering process. Manual removal or stringent selection criteria before clustering can largely eliminate this potential bias. Addition of new keyword thesauri such as species, technologies, drugs, toxicology, pathology, etc. is feasible. Pre-calculation of co-publication of all possible gene-gene and gene-keyword pairs and storage in the pairstat data file, makes querying the database extremely efficient. Although the data are present, CoPub Mapper is not programmed for co-occurrence querying of more than 2 concepts. We are currently integrating CoPub Mapper into the Sequence Retrieval System (SRS) for multi-concept interrogation and direct linkage to other databases (such as microarray data, Gene Ontology, OMIM, SwissProt, LocusLink, UniGene, Ensembl, etc.) [48].

Comparing the gene expression profiles of normal versus PCOS ovaries has identified a large number of genes representing networks and pathways that are deregulated in PCOS. However, the gene names and symbols hardly ever point to specific signal transduction pathways. The relation of genes with their function, localization and context has been described in literature. Here we show that within the list of differentially expressed genes some are linked to PCOS, obesity, diabetes and gametogenesis. This is without surprise and easily explained [46,47]. Other genes are linked to cell proliferation, differentiation and cancer. Most of them were downregulated which correlates with the observed arrest in growth and differentiation of follicles. Other clusters with no obvious link to PCOS may shed new light on the genes and pathways involved in the disease.

One of the major challenges associated with compiled heterogeneous text records such as MEDLINE, is correct gene recognition and assignment. The lack of consistent gene naming has resulted in a flood of synonyms and homonyms [7]. Although the synonym issue can be resolved by accumulating all different gene names and symbols, the correction for homonyms is still a daunting task. In order to include different spelling forms and the word context, we performed the text searches case insensitive and with predefined rules of regular expression.

The homonym problem consists of (i) different genes with identical gene name, symbol, or alias, and (ii), more frequently, a gene name, symbol or alias used for other terms than genes [9]. In the curated CoPub Mapper gene thesaurus, 1,286 of the 15,621 annotated genes (8.2 %) share a symbol or alias. In order to limit both aspects of the homonym problem, we (i) eliminated 2 letter symbols and aliases, (ii) deleted all symbols and aliases present in the English dictionary, (iii) manually curated terms with exceptionally high number of hits, (iv) corrected for cell line names, and (v) deleted records in which the preceding description of parenthesised symbols or aliases did not match the corresponding gene name. This last method has been used before to make an inventory of the homonym problem and provide strategies for correction, such as the one used here [9-13]. Although these measures effectively reduced the homonym problem, one will regularly encounter incorrect record assignment and invalid co-occurrence quotation using CoPub Mapper. Additional optimisation of the gene thesaurus might further reduce this problem to some extent, but other correction approaches should be considered. One of the most promising strategies to achieve disambiguation is based on the preferential co-occurrence of other concepts [9,10]. For example, concepts generally co-published with PSA meaning Poultry Science Association, will be very different from concepts co-published with PSA representing prostate specific antigen. Based on these preferential co-occurring concepts, one can assign the correct meaning to an ambiguous term.

Besides disclosure, summary, and visualisation of known facts using co-publication, one could also discover novel linkages among genes and between genes and other concepts. One possibility to identify unpublished, but plausible links, is to screen for black squares surrounded by red ones in a clustered co-occurrence heat map as shown in Figure 5. The fact that a particular gene-disease combination was not found in MEDLINE (black square), but clustered together with other co-published gene-disease pairs (red squares), could indicate an unpublished association. This approach shows analogies with the Swanson discovery framework in which concept A is known to relate to B and B is associated with C [49,50]. Combining all data, the deduction that A relates to C can be hypothesised and tested [49,51-53].

## Conclusion

CoPub Mapper is a program that identifies and rates co-published genes and keywords starting from a single concept search or batch-wise from a set of genes. Its modularity and pre-calculated co-occurrences allow for quick and versatile querying. The regular-expression search strategy and homonym correction makes the keyword database comprehensive and less contaminated with false positive classifications. CoPub Mapper can be used to summarize, evaluate and categorise annotated genes from microarray analyses based on co-occurrences with biological keywords and other published genes.

## Availability and requirements

The CoPub Mapper program is available for free use at this URL:  or 

## Authors' contributions

GJ, SvB, and JP conceived the approach and participated in the early design. BTFA and AV developed and optimised the software. TR developed and performed the homonym correction algorithm. RJ performed and interpreted the AUC ROC analyses and SV performed the MEDLINE gene and keyword searches. The project was supervised by GJ, TR and JP. All authors read and approved the final manuscript.

## Figures and Tables

**Table 1:** CoPub Mapper gene and keyword database information. Gene names, symbols and aliases were retrieved from Affymetrix HG_U95 / HG_U133 [54] and the HUGO databases [55]. The keyword thesauri include the three Gene Ontology subsections [41], diseases [56] and tissues/organs [57].

**Thesaurus**	**Data Source**	**Number of terms**	**Number of terms with MEDLINE hits**	**Total number of MEDLINE citations**
Gene	Affymetrix HG_U95-133 HUGO	15,621	10,700	5,932,448
Molecular Function	Gene Ontology	962	851	6,616,546
Cellular Component	Gene Ontology	218	196	1,890,561
Biological Process	Gene Ontology	767	621	3,455,950
Diseases	Karolinska Institute	1475	1444	6,099,280
Tissues	National Library of Medicine	309	307	9,083,831

**Table 2:** CoPub Mapper test groups. Eight groups of genes with a common function, process, cellular location, or microarray expression profile, were defined from gene ontology (GO), BioCarta, or a microarray experiment. The genes used for CoPub Mapper analysis were randomly selected from larger sets of genes part of the 8 different groups.

**Test groups**	**# Genes**	**Source**
smooth muscle contraction	12	GO (Biological Process)
acetyltransferase	18	GO (Molecular Function)
nuclear pore	15	GO (Cellular Component)
nucleosome	17	GO (Cellular Component)
ubiquitin	24	GO (Molecular Function)
hypoxia	26	GO (Biological Process)
BRCA1	11	BioCarta
Epithelial-specific genes	27	UniGEM V microarray: stroma vs epithelial cells

**Table 3:** CoPub Mapper single gene pair output. Output of the "Single Gene Pair Mapper" in which the top ten genes co-published with the androgen receptor are listed according to number of co-publications (Pmid hits).

**Gene Name**	**Gene Symbols**	**Gene Alias**	**Pmid Hits**
progesterone receptor	PGR	NR3C3	605
kallikrein 3, prostate specific antigen	KLK3	PSA	414
nuclear receptor subfamily 3, group C, member 1; glucocorticoid receptor	NR3C1	GCR, GRL	389
aminopeptidase puromycin sensitive	NPEPPS	MP100, PSA	246
sex hormone-binding globulin	SHBG	ABP	179
gonadotropin-releasing hormone 1, leutinizing-releasing hormone	GNRH1	GNRH, GRH, LHRH, LNRH	157
prolactin	PRL		131
insulin	INS		125
epidermal growth factor, beta-urogastrone	EGF	URG	123
tumor protein p53	TP53	P53	94

**Table 4:** CoPub Mapper single gene biological concept output. Output of the "Single Gene Biological Term Mapper" in which the top ten diseases co-published with the androgen receptor are listed according to their relevance score.

**Keywords**	**Number of hits**	**log10 Relative Score**
Androgen-Insensitivity Syndrome	229	3.07
Kennedy Disease	21	2.56
Muscular Atrophy Spinal	133	2.12
Prostate Cancer	932	1.93
Gynecomastia	59	1.88
Hypospadia	81	1.79
Sex Chromosome Aberrations	2	1.78
Hirsutism	76	1.78
Robinow Syndrome	2	1.71
X-Linked Myotubular Myopathy	2	1.65

**Table 5:** CoPub Mapper single gene biological concept output. Output of the "Single Gene Biological Term Mapper" in which the top ten genes co-published with the prostate cancer disease-keyword are listed according to number of co-publications.

**Gene name**	**Gene Symbols**	**Gene Aliases**	**Number of hits**	**log10 Relative Score**
kallikrein 3, prostate specific antigen	KLK3	PSA	6628	2.55
aminopeptidase puromycin sensitive	NPEPPS	MP100, PSA	4507	2.57
androgen receptor, dihydrotestosterone receptor		DHTR, NR3C4	932	1.93
acid phosphatase, prostate	ACPP		546	2.22
gonadotropin-releasing hormone	GNRH1	GNRH, GRH, LHRH, LNRH	522	1.24
1, leutinizing-releasing hormone tumor protein p53	TP53	P53	431	0.96
B-cell CLL/lymphoma 2	BCL2		346	1.17
insulin	INS		318	0.05
epidermal growth factor, beta- urogastrone	EGF	URG	251	0.72
cyclin-dependent kinase inhibitor 1A	CDKN1A	CAP20, CDKN1, CIP1, MDA-6, P21, SDI1, WAF1	190	0.98
